# Synthesis and Discovery of Ligustrazine–Heterocycle Derivatives as Antitumor Agents

**DOI:** 10.3389/fchem.2022.941367

**Published:** 2022-07-25

**Authors:** Shitang Ma, Ning Zhang, Jiafu Hou, Shijuan Liu, Jiawen Wang, Baowei Lu, Fucheng Zhu, Peipei Wei, Ge Hong, Tianjun Liu

**Affiliations:** ^1^ College of Biological and Pharmaceutical Engineering, West Anhui University, Lu’an, China; ^2^ College of Life and Health, Anhui Science and Technology University, Fengyang, China; ^3^ Institute of Biomedical Engineering, Chinese Academy of Medical Sciences and Peking Union Medical College, Tianjin Key Laboratory of Biomedical Material, Tianjin, China; ^4^ Mudanjiang Medical University, Mudanjiang, China

**Keywords:** ligustrazine–heterocyclic (TMPH) derivatives, triple-negative breast cancer, antitumor, proliferation, apoptosis

## Abstract

Ligustrazine (TMP) is a natural pyrazine alkaloid extracted from the roots of Ligusticum Chuanxiong Hort, which has the potential as an antitumor agent. A series of 33 ligustrazine–heterocycle (TMPH) derivatives were designed, synthesized, and investigated via antitumor screening assays, molecular docking analysis, and prediction of drug-like properties. TMP was attached to other heterocyclic derivatives by an 8–12 methylene alkyl chain as a linker to obtain 33 TMPH derivatives. The structures were confirmed by ^1^H-NMR, ^13^C-NMR, and high-resolution mass spectroscopy spectral (HR-MS) data. The antiproliferative activity against human breast cancer MCF-7, MDA-MB-231, mouse breast cancer 4T1, mouse fibroblast L929, and human umbilical vein endothelial HUVEC cell lines was evaluated by MTT assay. Compound **12–9** displayed significant inhibitory activity with IC_50_ values in the low micromolar range (0.84 ± 0.02 µM against the MDA-MB-231 cell line). The antitumor effects of compound **12–9** were further evaluated by plate cloning, Hoechst 33 342 staining, and annexin V-FITC/PI staining. The results indicated that compound **12–9** inhibited the proliferation and apoptosis of breast cancer cells. Furthermore, molecular docking of compound **12–9** into the active site of the Bcl-2, CASP-3, and PSMB5 target proteins was performed to explore the probable binding mode. The 33 newly synthesized compounds were predicted to have good drug-like properties in a theoretical study. Overall, these results indicated that compound **12–9** inhibited cell proliferation through PSMB5 and apoptosis through Bcl-2/CASP-3 apoptotic signaling pathways and had good drug-like properties. These results provided more information, and key precursor lead derivatives, in the search for effective bioactive components from Chinese natural medicines.

## Introduction

Breast cancer is one of the most diagnosed malignant tumors in women worldwide, with high morbidity and mortality (Jaaks et al., 2022; Sammut et al., 2022). Triple-negative breast cancer (TNBC) is the most aggressive subtype of breast cancer (El-Arabey and Abdalla, 2022; Yang et al., 2022; Zhang S. et al., 2022) and is characterized by large tumor size, high content of positive lymph nodes, high histological grade with high mortality, and high recurrence and metastasis rates (Xiu et al., 2022). TNBC is one of the main causes of cancer death in women worldwide (Altei et al., 2022). The current strategies for the treatment of TNBC include radiotherapy (Raafat Elsayed et al., 2022), chemotherapy, and targeted therapy (Mao et al., 2021). The treatment of TNBC with natural medicines has also been investigated. Despite remarkable advances in the treatment of TNBC (Chen et al., 2022), the lack of efficacious anticancer drugs is still a problem (Zhang P. et al., 2022). In addition, the side effects of existing drugs are a major obstacle in the treatment of TNBC (Olivier and Prasad, 2022; Zhang D. et al., 2022). It is therefore desirable to develop new effective anticancer drugs for the treatment of TNBC.

Proteasomes play a critical role in nuclear and cytosolic proteolysis (Dwivedi et al., 2021; Jia et al., 2021), and the inhibition of proteasomes has been proposed as an effective strategy against TNBC (Ramdas et al., 2019). The proteasome 20S subunit beta 5 (PSMB5) (Liu et al., 2020), which is a key regulator of proteasome function (Wei et al., 2018), is a promising target protein for proteasome inhibition in the treatment of TNBC (Liu et al., 2020). Pathways related to PSMB5 include signaling by Hedgehog, mitotic G1-G1/S phases (Oerlemans et al., 2008), and down-regulation by IFN-gamma (at the protein level) (Rut and Drag, 2016). The high expression of PSMB5 has been shown to be indicative of poor prognosis in TNBC patients (Liu et al., 2020). Silencing of PSMB5 sensitized cells to apoptosis and ultimately enhanced the effect of chemotherapy (Wei et al., 2018; Adwal et al., 2020). PSMB5 has been associated with cancer cell proliferation in TNBC patients (Liu et al., 2020). As signaling pathways that regulate cell apoptosis and survival (Guan et al., 2022), Bcl-2 and CASP-3 apoptotic signaling pathways have also been implicated in TNBC by promoting, or inhibiting, apoptotic pathways triggered by mitochondrial dysfunction (Shen et al., 2020; An et al., 2021; Guan et al., 2022). The development of a potent anticancer agent that is capable of inhibiting proliferation through PSMB5 and apoptosis through Bcl-2/CASP-3 apoptotic signaling pathways has emerged as an effective strategy for TNBC treatment (Lang et al., 2019; Nabil et al., 2021). PSMB5, Bcl-2, and CASP-3 play key roles in the signaling pathway of TNBC, and these proteins are attractive targets in the development of ligand molecules as potential antitumor agents. Many compounds have been designed, synthesized, and evaluated as inhibitors of these target proteins. ABT-199 and derivatives with an acyl sulfonamide skeleton were shown to have antiproliferative effects targeting Bcl-2 (Wang et al., 2021). Bortezomib, carfilzomib, and syringolin analogs have demonstrated potential antitumor activity targeting PSMB5 (Yoshida et al., 2018; Allmeroth et al., 2021). Furthermore, compound 1,2,3-triazole-thiazolidinone-carvone hybrid derivatives showed moderate antiproliferative activity through the CASP-3 pathway (Oubella et al., 2021). Lead compounds, including curcumin, quercetin, and baicalin, from natural medicines, have also shown antitumor activity by regulating the signaling pathways of PSMB5, Bcl-2, and CASP-3 (Chen et al., 2018; Mortezaee et al., 2019).

Bioactive components from natural medicines, a key source of lead compounds for the development of innovative drugs (Kataura et al., 2021), have a wide range of biological activities (Jiang et al., 2021), including antitumor, anti-inflammatory, and anti-infection activity. Many antitumor agents, such as vinblastine, paclitaxel, and ligustrazine (TMP), have been isolated from natural products. Natural medicines can have additive or synergistic effects (Yang et al., 2021) by simultaneously acting on multiple targets of the TNBC pathway. Extracts of *Rosmarinus officinalis* L., *Astragalus* polysaccharides, and bioactive components of tetrandrine are useful in the treatment of TNBC because these compounds have the ability to improve the postoperative “tumor constitution” (Chen et al., 2021). TMP is a natural alkaloid monomer pyrazine derivative extracted and isolated from the roots of Ligusticum Chuanxiong Hort, which has been shown to be a potential antitumor agent (Xie et al., 2019). TMP has demonstrated multiple antitumor effects, including inhibiting proliferation (Luo et al., 2021), apoptosis, invasion, and metastasis of malignant tumors (Bian et al., 2021). The introduction of substitutions into the TMP nucleus may improve the antitumor effect (Xu et al., 2017a; Wang et al., 2019). A TMP dimer with an alkyl chain as the linker had significantly stronger antitumor activity than the TMP monomer (Li et al., 2014). TMP-chalcone hybrids demonstrated antiproliferative activity in an *in vivo* model of TNBC (Luo et al., 2021).

In the present study, flexible alkyl chains of different lengths (8–12 carbons) were used as linkers. TMP was structurally combined with other pharmaco-active heterocycles, according to the splicing method of active substructures, to synthesize new ligustrazine–heterocycle (TMPH) derivatives. A series of 33 TMPH derivatives were designed, synthesized, and investigated via antitumor screening assays, molecular docking with Bcl-2, CASP-3, and PSMB5 target proteins, and prediction of drug-like properties. The structures of the newly synthesized derivatives were confirmed by ^1^H-NMR, ^13^C-NMR, and HR-MS spectral data. The antiproliferative activity against five cell lines (human breast cancer MCF-7 and MDA-MB-231, mouse breast cancer 4T1, mouse fibroblast L929, and human umbilical vein endothelial HUVEC) was evaluated by MTT assay. In addition, one of the TMPH derivatives with potent antiproliferative activity was selected for further evaluation by plate cloning, Hoechst 33 342 staining, and annexin V-FITC/PI staining. Molecular docking into the active site of the Bcl-2, CASP-3, and PSMB5 target proteins was performed to explore the probable binding mode. Finally, the 33 newly synthesized compounds were predicted to have good drug-like properties in a theoretical study.

## Materials and Methods

The experimental section contains seven main sections as follows:

• Synthesis and Structure Elucidation of the 33 TMPH Compounds
• *In Vitro* Antiproliferative Activity Evaluation by MTT Assay
• Clonal Formation Assay of MDA-MB-231 Cells
• Hoechst 33 342 Staining of MDA-MB-231 Cells
• Apoptosis Analysis by Annexin V-FITC/PI Staining
• Molecular Docking to Bcl-2, CASP-3, and PSMB5
• Prediction of *In Silico* Drug-Like Properties

### Synthesis and Structure Elucidation of the 33 TMPH Compounds

All solvents and reagents used were obtained from commercial sources and used without further purification. TMP was provided by Aladdin^®^ (Shanghai, China) with a purity of 98% as indicated by HPLC. The progress of the synthetic reactions was monitored by thin-layer chromatography (TLC) on silica gel GF254 plates, and the target products were purified by silica gel column chromatography using a mixture of petroleum ether and acetone as the eluent. The purity of the target compounds was >95% as determined by HPLC (Waters 2695 with a Kromasil C18 column eluted by methanol/water 85:15). The accurate mass was measured using a VG ZAB-HS double-focusing magnetic mass spectrometer. ^1^H-NMR and ^13^C-NMR spectra were collected using a Bruker 400 MHz superconducting Fourier transform liquid NMR spectrometer and Avance III 400 MHz liquid NMR spectrometer, using tetramethylsilane and DMSO-*d*_*6*_ as the internal reference and solvent, respectively. The chemical shifts of the protons were recorded in ppm (δ). The structure characterization data for the 33 new TMPH compounds are shown in detail in the Supplementary Material.

### *In Vitro* Antiproliferative Activity Evaluation by MTT Assay

The *in vitro* antiproliferative activity of 33 TMPH compounds was evaluated by MTT assay (Medica et al., 2022). Five types of TNBC-related cell lines (human breast cancer MCF-7 and MDA-MB-231, mouse breast cancer 4T1, mouse fibroblast L929, and human umbilical vein endothelial HUVEC cell lines) were used. The cell lines were obtained from the Shanghai Institute of Biochemistry and Cell Biology of the Chinese Academy of Sciences (Shanghai, China). The cell lines were cultured in DMEM supplemented with 1% penicillin–streptomycin solution (100×) and 10% FBS at 37°C under a humid atmosphere containing 5% CO_2_. After cell passage, cryopreservation, and resuscitation, the growth-inhibitory effects against the five cell lines were determined by MTT assay. The 33 TMPH compounds and the reference control drug cisplatin were dissolved in 0.1% DMSO solution. The cell lines were maintained in 96-well plates and subjected to each test compound (0–50 μM) at 37°C for 48 h. After treatment with 1 mg/ml MTT solution for 4 h, the formazan crystals were dissolved in each well. A microplate reader (Varioskan Flash 3001, Thermo Scientific) was used to determine the cell viability at 490 nm, and each experiment was repeated four times. The collected data were expressed as the mean ± SEM. The inhibitory concentration of the 34 compounds at 50% (IC_50_) was calculated via concentration–inhibition relationship regression by SPSS software.

### Clonal Formation Assay of MDA-MB-231 Cells

Human breast cancer MDA-MB-231 cells were cultured in a six-well plate with 1,500 cells per well and incubated for 24 h at 37°C under a humid atmosphere containing 5% CO_2_. Then, the MDA-MB-231 cells were incubated with compound **12–9** at final concentrations of 0, 0.5, 1.0, and 2.0 μM for 10 days. The treated cells were washed with PBS and fixed with 4% paraformaldehyde for 15 min at room temperature. Then, the end product of the cell colonies was visualized by staining with 0.1% crystal violet for 10 min. The images were photographed using a handy camera, and the number of cell colonies was assessed using open Image J software (National Institutes of Health). Each experiment was repeated three times. A group of >50 cells was defined as one colony formation.

### Hoechst 33 342 Staining of MDA-MB-231 Cells

The induction of apoptosis by compound **12–9** was visually evaluated in MDA-MB-231 cells by Hoechst 33 342 staining. The test cells were transferred to six-well plates at a density of 1 × 10^5^ cells. After incubation for 24 h, 2 ml of media containing 2 μM compound **12–9** was used to replace the culture medium. Then, the MDA-MB-231 cells were stained with Hoechst 33 342 solution (10 μg/ml in the culture medium, Beyotime Institute of Biotechnology, Shanghai, China) at 37°C in a dark room for 20 min after incubation with the test compound **12–9**. The cells were then washed twice with a serum-free medium to remove the leftover dye. The apoptotic cells stained by Hoechst 33 342 were imaged using CLSM (Carl-Zeiss LSM 710).

### Apoptosis Analysis by Annexin V-FITC/PI Staining

Apoptosis induced by TMPH compound **12–9** was evaluated by annexin V-FITC/PI staining. Briefly, MDA-MB-231 cells in the logarithmic growth phase were cultured in a six-well plate and incubated for 24 h at 37°C under a humid atmosphere containing 5% CO_2_. Then, the cultured cells were incubated with TMPH **12–9** at final concentrations of 0, 0.5, 1.0, and 2.0 μM for 24 h. The incubated cells were washed twice with PBS, digested by trypsin, and centrifuged at 1,000 rpm for 5 min. The collected MDA-MB-231 cells were transferred to a 1.5-ml tube and resuspended in a solution of 200 μl of annexin V-FITC and 5 μl of PI. After incubation in a dark environment (covered with aluminum foil) for 15 min at room temperature, the collected cells were evaluated by flow cytometry (CyFlow®Cube 6, Sysmex). The apoptosis analysis data were shown as a pseudo color map and analyzed by FlowJo software.

### Molecular Docking to Bcl-2, CASP-3, and PSMB5

Molecular docking analysis is widely used to evaluate the potential binding affinity and interaction mechanism between a ligand and a target protein (Ma et al., 2021). To further elucidate the mechanism of action (Zhang et al., 2020), compound **12–9** was subjected to flexible receptor docking with the proteins, Bcl-2, CASP-3, and PSMB5, using the molecular docking protocols of the Schrodinger Maestro platform (https://www.schrodinger.com/). The crystal structures obtained by X-ray crystallography with the highest resolution in the form of the lowest value were selected for the docking study. The originated ligands for each target are given here as a reference. The crystal structures of the targets Bcl-2 Bcl-2 (PDB ID: 6GL8), CASP-3 (PDB ID: 1NME), and PSMB5 (PDB ID: 5LEY) were retrieved from RCSB PDB (https://www.pdbus.org/) and subjected to the protein preparation wizard. A glide grid box was then generated around the original ligand inhibitor in the binding pocket of each target protein (Ma et al., 2018). The glide ligand docking module was then used to conduct between the ligand compounds and the proteins Bcl-2, CASP-3, and PSMB5. The binding mode for a given ligand with each target protein was identified by the highest binding energy in the form of a docking score. Only flexibility was taken into account, and the protein was considered to form a semi-rigid pocket. The docking results for the ligand–protein complexes were obtained according to the docking scores, which were calculated using the Schrodinger Maestro scoring function.

### Prediction of *In Silico* Drug-Like Properties

Poor drug-like properties can lead to the failure of a potential drug (Ji, 2021), and the prediction of drug-like properties is becoming more critical in the development of lead compounds (Gupta et al., 2021). Also, 12 mathematical models for the molecular descriptors nHA (number of hydrogen bond acceptors), nHD (number of hydrogen bond donors), TPSA (topological polar surface area), MW (molecular weight), logP (logarithm of the *n*-octanol/water distribution coefficient), PPB (plasma protein binding), VD (volume distribution), CL (clearance), T_1/2_ (half-life), and IGC_50_ (48 h *Tetrahymena pyriformis* IGC_50_ value), which characterize the physicochemical, medicinal chemistry, and ADMET properties, were calculated by Schrodinger Canvas and web-based applications of ADMETlab (https://admetmesh.scbdd.com/). The drug-like properties of the 33 TMPH compounds, as well as the TMP reference, were then evaluated.

## Results and Discussion

### Synthesis and Structure Elucidation of the 33 TMPH Compounds

**SCHEME 1:**
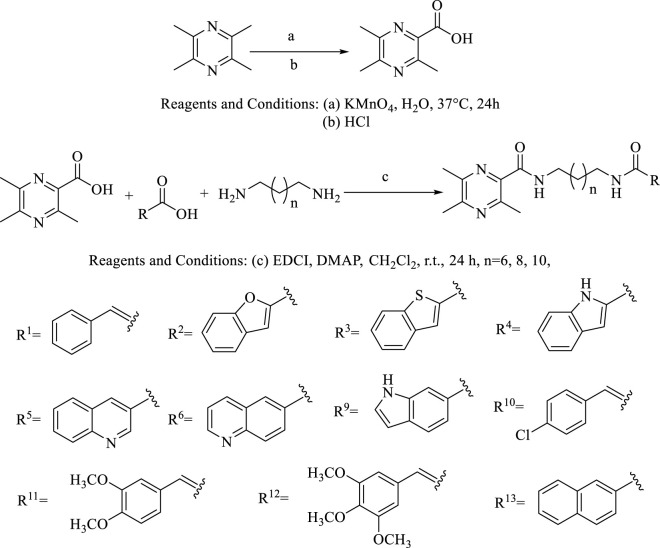
Synthetic route for the TMPH derivatives.

The general synthetic procedures for the target products are outlined in Scheme 1. The intermediate TMP-COOH was first synthesized by the treatment of the precursor TMP with KMnO_4_ and HCl in water at 37°C for 24 h. The target compounds linked by an amide chain were synthesized by a reaction with 1-(3-dimethylaminopropyl)-3-ethylcarbodiimide hydrochloride (EDCl) and 4-dimethylaminopyridine (DMAP) in anhydrous CH_2_Cl_2_ at room temperature. To explore the effect of the length of the linker, TMP moieties with an 8–12 methylene alkyl chain as a linker were synthesized. All the target compounds were characterized by ^1^H-NMR, ^13^C-NMR, and HR ESI-MS. The details of the physical properties and the spectral data are provided in the Supplementary Material.

### *In Vitro* Antiproliferative Activity Evaluation by MTT Assay

The antiproliferative activity of the compounds was investigated against five cell lines (human breast cancer MCF-7 and MDA-MB-231, mouse breast cancer 4T1, mouse fibroblast L929, and human umbilical vein endothelial HUVEC). The ability of the synthesized compounds to inhibit cell proliferation in the five cell lines was evaluated by MTT assay with cisplatin as a positive control. The IC_50_ values for the antiproliferative activity of the compounds are shown in Table 1. The biological activity of all the TMPH derivatives was higher than that of the original TMP against the tested five cell lines. All the compounds displayed moderate to good inhibition against MDA-MB-231 cells, compared with the reference drug, with IC_50_ values ranging from 0.84 ± 0.02 to 13.02 ± 0.27 µM. In addition, 42.42% (14/33) of the compounds (compounds **8–3**, **8–10**, **10–6**, **10–9**, **10–13**, **12–1**, **12–2**, **12–3**, **12–5**, **12–6**, **12–9**, **12–10**, **12–12**, and **12–13**) had broad-spectrum cytotoxic activity in all tested cell lines with IC_50_ values ranging from 1.61 ± 0.08 to 19.47 ± 0.45 µM. The antiproliferative activity increased with increasing carbon chain length in the flexible linker, and the activity was the highest with a 12-carbon alkyl chain linker. In addition, compounds **10–9, 12–3**, and **12–9** had higher activity than cisplatin in the MCF-7, 4T1, and L929 cell lines, and the selective index (SI) values between L929 and MDA-MB-231 [IC_50_ (L929)/IC_50_ (MDA-MB-231)] were 1.23, 3.61, and 3.76, respectively. In contrast, the SI value for cisplatin was 2.64. Compound **12–9** with the 12-carbon alkyl chain, 1,12-diaminododecane, displayed notable antiproliferative activity compared with the reference drug cisplatin and the other TMPH compounds, which had IC_50_ values ranging from 0.84 ± 0.02 to 12.37 ± 0.09 µM against the five cell lines. These results encouraged us to further investigate the possible antitumor mechanisms of TMPH **12–9**. Structure–activity relationship analysis suggested that the introduction of a heterocyclic ring increased the antiproliferative activity of the TMPH compounds to a certain extent. The *in vitro* antiproliferative activity increased with the increasing carbon chain length in the linker. In the derivatives with a 12-carbon chain, the introduction of a chlorine group had no obvious effect (**12–10** vs. **12–1**). The cytotoxicity toward the MDA-MB-231 cell lines increased with an increasing number of methoxy groups (**12–12** vs. **12–11** with three and two methoxy groups, respectively). In addition, the SI value increased with the introduction of a benzimidazole group replacing the benzene group (**12–4** vs. **12–1**). Compared with compound **12–4**, compound **12–9** showed much promising antiproliferative activity in most of the tested cell lines. The structures of **12–4** and **12–9** are very similar, but the position of the carboxylic acid substituent plays a key role in the antiproliferative activity. No obvious differences in the activity in most of the tested cell lines were found between the three compounds with two fused six-membered rings (**12–5**, **12–6**, and **12–13**). These results indicated that the introduction of a nitrogen atom into the fused six-membered rings had no appreciable effect on MTT activity. Many TMP derivatives have been synthesized and evaluated with potent antitumor activity, including monocarbonyl TMP–curcumin hybrids (Ai et al., 2016), TMP–betulinic acid hybrids (Xu et al., 2017b), and TMP–rhein derivatives. A TMP dimer linked by decane-1,10-diamine exhibited cytotoxicity in FaDu cells with an IC_50_ value of 1.36 nM (Wang et al., 2019), and a TMP–vanillic acid amide derivative had displayed high potency with an EC_50_ value of 17.39 μM (Xu et al., 2017a). In the present study, TMPH derivatives with a flexible alkyl chain were developed with IC_50_ values in the low micromolar range.

**TABLE 1:** Cytotoxicity of ligustrazine–heterocyclic derivatives (TMPH) to five kinds of cells by MTT assay.

TMPH	Antiproliferative activity IC_50_ ± SEM (µM)					
	MCF-7	MDA-MB-231	4T1	L929	HUVEC	SI
**8–1**	10.99 ± 0.06	2.52 ± 0.08	>20	16.58 ± 0.12	>20	6.58
**8–2**	6.62 ± 0.15	6.10 ± 0.16	19.86 ± 0.16	>20	4.27 ± 0.07	>3.28
**8–3**	4.15 ± 0.02	3.39 ± 0.12	19.47 ± 0.45	4.12 ± 0.32	9.78 ± 0.05	1.22
**8–4**	6.21 ± 0.14	5.77 ± 0.09	>20	10.70 ± 0.43	>20	1.85
**8–5**	4.96 ± 0.25	3.44 ± 0.16	2.07 ± 0.04	3.49 ± 0.28	>20	1.01
**8–6**	>20	1.02 ± 0.15	1.94 ± 0.03	>20	>20	>19.61
**8–9**	>20	8.45 ± 0.14	3.74 ± 0.19	>20	>20	>2.37
**8–10**	2.84 ± 0.21	6.67 ± 0.16	2.48 ± 0.24	5.48 ± 0.06	4.54 ± 0.01	0.82
**8–11**	>20	5.05 ± 0.02	11.95 ± 0.46	>20	10.35 ± 0.03	>3.96
**8–12**	5.38 ± 0.13	11.24 ± 0.12	10.25 ± 0.32	>20	>20	>1.78
**8–13**	>20	2.44 ± 0.09	13.68 ± 0.24	15.67 ± 0.36	14.40 ± 0.03	6.42
**10–1**	2.61 ± 0.19	1.14 ± 0.08	2.86 ± 0.18	4.36 ± 0.37	>20	3.82
**10–2**	4.19 ± 0.15	8.41 ± 0.18	>20	11.66 ± 0.81	16.63 ± 0.68	1.39
**10–3**	18.54 ± 1.34	4.16 ± 0.25	17.55 ± 1.16	7.26 ± 0.16	>20	1.75
**10–4**	>20	13.02 ± 0.27	>20	>20	>20	>1.54
**10–5**	8.72 ± 0.49	5.76 ± 0.13	>20	5.18 ± 0.27	>20	0.90
**10–6**	6.49 ± 0.36	7.76 ± 0.14	11.98 ± 0.54	8.91 ± 0.12	10.21 ± 0.86	1.15
**10–9**	3.37 ± 0.19	6.01 ± 0.11	3.12 ± 0.14	7.37 ± 0.57	9.63 ± 0.53	1.23
**10–10**	2.93 ± 0.05	2.83 ± 0.09	>20	10.13 ± 0.52	11.16 ± 0.38	3.58
**10–11**	3.23 ± 0.03	12.89 ± 1.41	>20	>20	>20	>1.55
**10–12**	2.86 ± 0.08	3.41 ± 0.12	>20	>20	>20	>5.87
**10–13**	3.09 ± 0.13	4.60 ± 0.29	9.88 ± 0.22	12.64 ± 0.12	10.51 ± 1.24	2.75
**12–1**	1.95 ± 0.06	2.78 ± 0.37	2.66 ± 0.05	4.75 ± 0.26	13.17 ± 1.12	1.71
**12–2**	2.71 ± 0.07	3.38 ± 0.12	3.97 ± 0.01	3.78 ± 0.14	10.96 ± 0.68	1.12
**12–3**	3.26 ± 0.16	2.12 ± 0.06	5.13 ± 0.28	7.65 ± 0.06	7.86 ± 0.01	3.61
**12–4**	3.23 ± 0.18	3.52 ± 0.12	6.62 ± 0.14	>20	>20	>5.68
**12–5**	1.36 ± 0.06	2.39 ± 0.12	3.50 ± 0.06	4.18 ± 0.05	11.36 ± 0.44	1.75
**12–6**	2.04 ± 0.03	1.98 ± 0.15	4.18 ± 0.09	5.35 ± 0.17	7.03 ± 0.05	2.70
***12–9***	1.11 ± 0.15	**0.84 ± 0.02**	1.16 ± 0.18	3.16 ± 0.13	3.82 ± 0.24	3.76
**12–10**	2.09 ± 0.06	2.42 ± 0.06	1.63 ± 0.03	3.82 ± 0.07	12.37 ± 0.09	1.58
**12–11**	1.76 ± 0.02	2.42 ± 0.18	2.32 ± 0.14	>20	>20	>8.26
**12–12**	2.21 ± 0.15	1.64 ± 0.12	2.69 ± 0.04	18.86 ± 0.44	12.16 ± 0.20	11.50
**12–13**	1.61 ± 0.08	5.01 ± 0.16	4.76 ± 0.45	8.89 ± 0.12	10.70 ± 1.06	1.77
TMP	>20	>20	>20	>20	>20	NC
DDP	6.25 ± 0.34	3.18 ± 0.06	5.71 ± 0.16	8.38 ± 0.23	7.46 ± 0.45	2.64

SI, selectivity index; SI = IC_50_ (L929)/IC_50_ (MDA-MB-231); NC, not calculated.

### Clonal Formation Assay of MDA-MB-231 Cells

**FIGURE 1:**
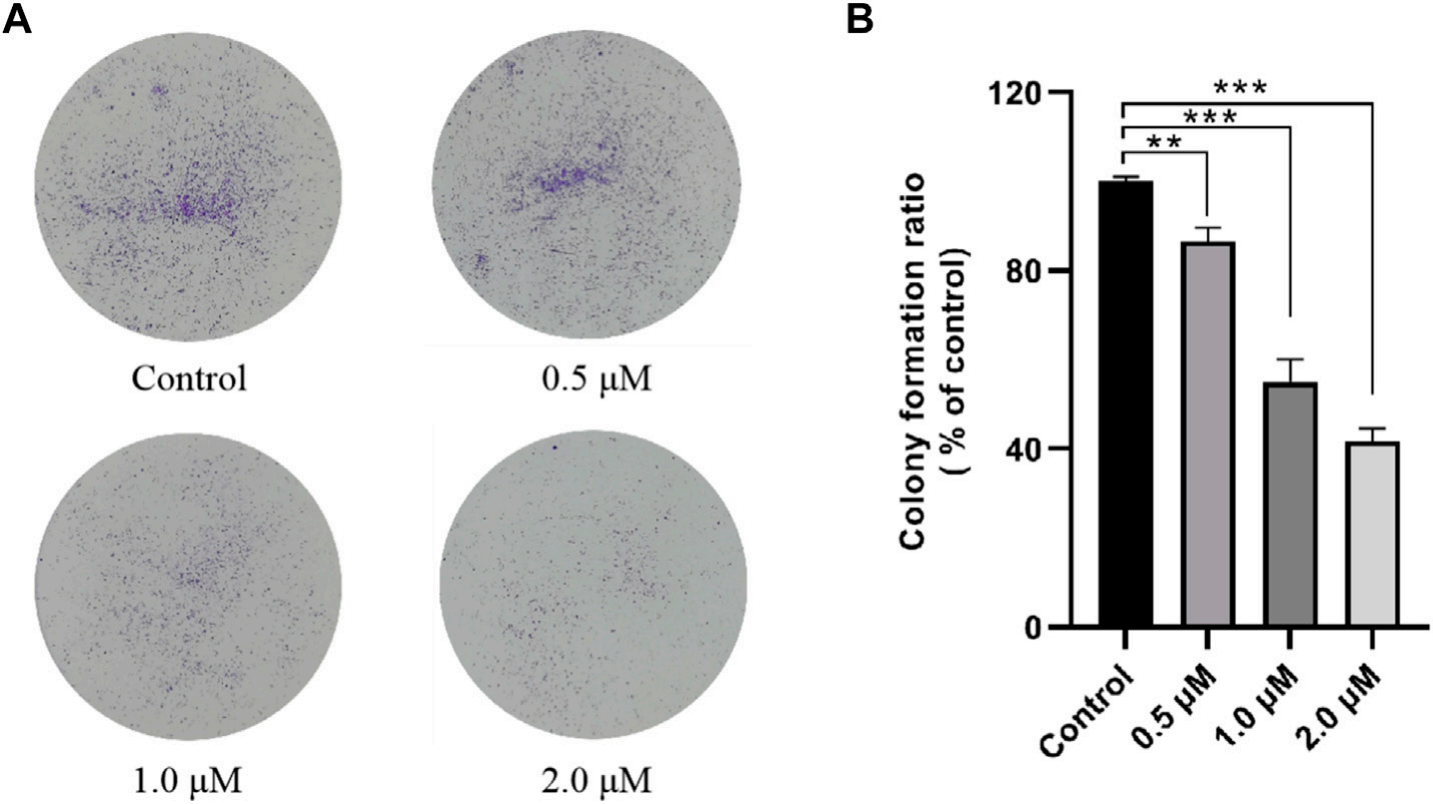
Effect of TMPH **12–9** on the colony formation in MDA-MB-231 cells. **(A)** Representative images of MDA-MB-231 colonies; **(B)** percentage of colonies compared with that of the control group, *n* = 3. ^∗∗^: *p* < 0.05; ^∗∗∗^: *p* < 0.001 versus control.

According to the MTT assay results shown in Table 1, compound **12–9** had the highest antiproliferative activity *in vitro*, especially in the human breast cancer MDA-MB-231 cell line (IC_50_ = 0.84 ± 0.02 µM) of all the tested compounds. Thus, compound **12–9** was selected for further investigation in the colony formation assay in MDA-MB-231 cells. As shown in Figure 1, in MDA-MB-231 cells treated with compound **12–9** at concentrations of 0.5, 1.0, and 2.0 μM for 10 days, the colonies formed by cloning were significantly fewer and smaller than the untreated control group. Compound **12–9** also exhibited dose-dependent inhibition of the proliferation of MDA-MB-231 cells. These results indicated that TMPH **12–9** reduced the clonogenic ability of MDA-MB-231 cells and inhibited the proliferation of tumor cells.

### Hoechst 33 342 Staining of MDA-MB-231 Cells

**FIGURE 2:**
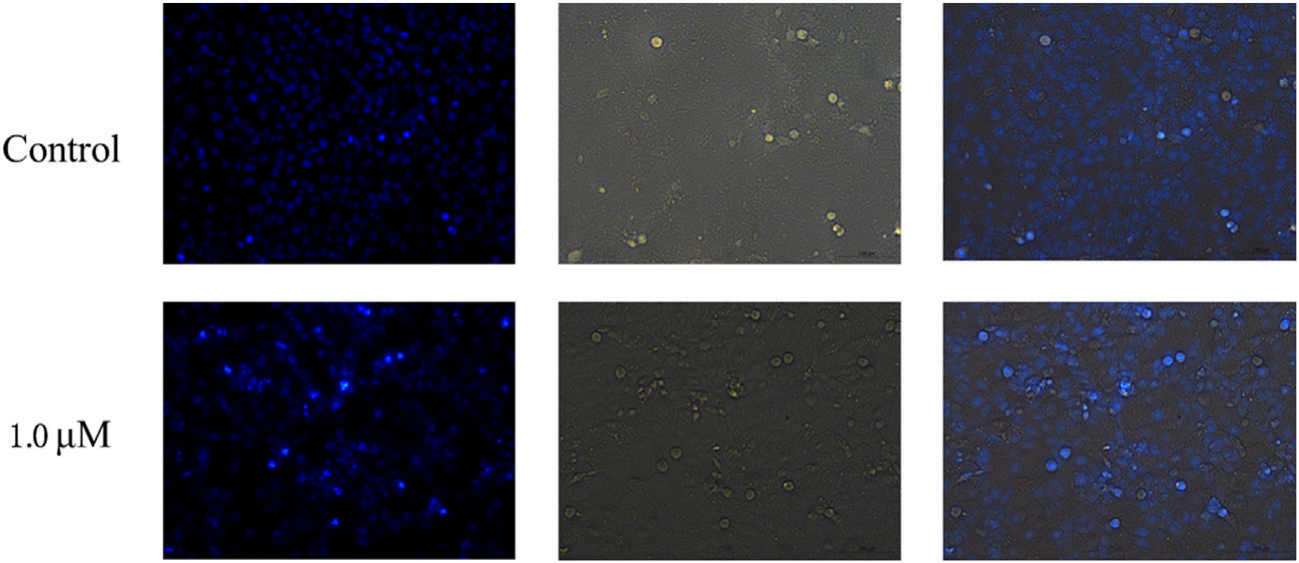
Fluorescence microscopy images of MDA-MB-231 cells with Hoechst staining: the control group and treatment group with 1.0 μM TMPH **12–9**.

The ability of compound **12–9** to induce apoptosis in MDA-MB-231 cells was investigated by Hoechst 33 342 staining. MDA-MB-231 cells were allowed to grow in the presence (1.0 μM) or absence of TMPH **12–9** for 48 h. As shown in Figure 2, the untreated MDA-MB-231 cells as the control group exhibited almost negligible apoptotic characteristics with uniform weak blue fluorescence. In contrast, most of the MDA-MB-231 cells in the TMPH **12–9** treatment group appeared shrunken and showed strong blue fluorescence. The aforementioned results suggested that the induction of apoptosis in MDA-MB-231 cells might be the cause of the cell growth inhibition observed for compound **12–9**.

### Apoptosis Analysis by Annexin V-FITC/PI Staining

**FIGURE 3:**
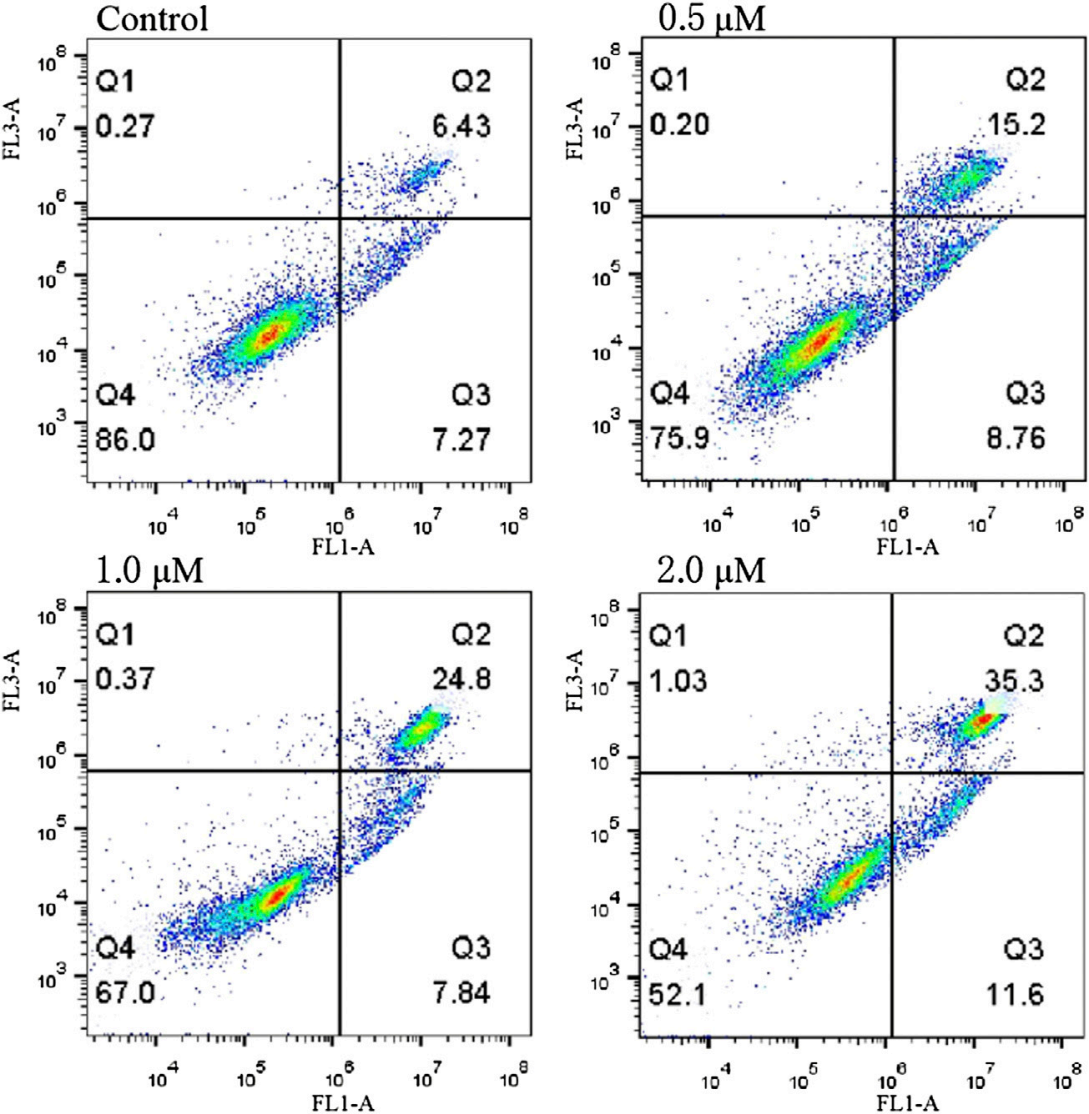
Annexin V-FITC and PI staining to evaluate apoptosis in MDA-MB-231 cells following compound **12–9** treatment. MDA-MB-231 cells were treated with MDA-MB-231 (0, 0.5, 1.0, and 2.0 μM, for 48 h), stained by annexin V-FITC and PI, and analyzed using flow cytometry.

The ability of compound **12–9** to induce apoptosis was evaluated by annexin V-FITC/PI flow cytometry. Human breast cancer MDA-MB-231 cells were allowed to grow in the presence (0.5, 1.0, and 2.0 μM) or absence of **12–9** for 48 h and then were co-stained with PI and annexin-V/FITC chromogenic agents. The quadrant images in Figure 3 represent four different cell states: Q1, necrotic cells (annexin V^−^/PI^+^); Q2, late apoptotic or necrotic cells (annexin V^+^/PI^+^); Q3, early apoptotic cells (annexin V^+^/PI^−^); and Q4, living cells (annexin V^−^/PI^−^). As shown in Figure 3, after treatment with different concentrations of compound **12–9** for 48 h, the apoptotic ratio in MDA-MB-231 cells increased gradually from 13.7% for the reference control group to 23.96% (0.5 μM **12–9**), 32.64% (1.0 μM **12–9**), and 46.9% (2.0 μM **12–9**). These data indicated that TMPH compound **12–9** effectively induced apoptosis in MDA-MB-231 cells in a dose-dependent manner, from 0.5 to 2.0 μM TMPH **12–9**. This result was consistent with the Hoechst 33 342 staining results for apoptosis analysis.

### Molecular Docking to Bcl-2, CASP-3, and PSMB5

**FIGURE 4:**
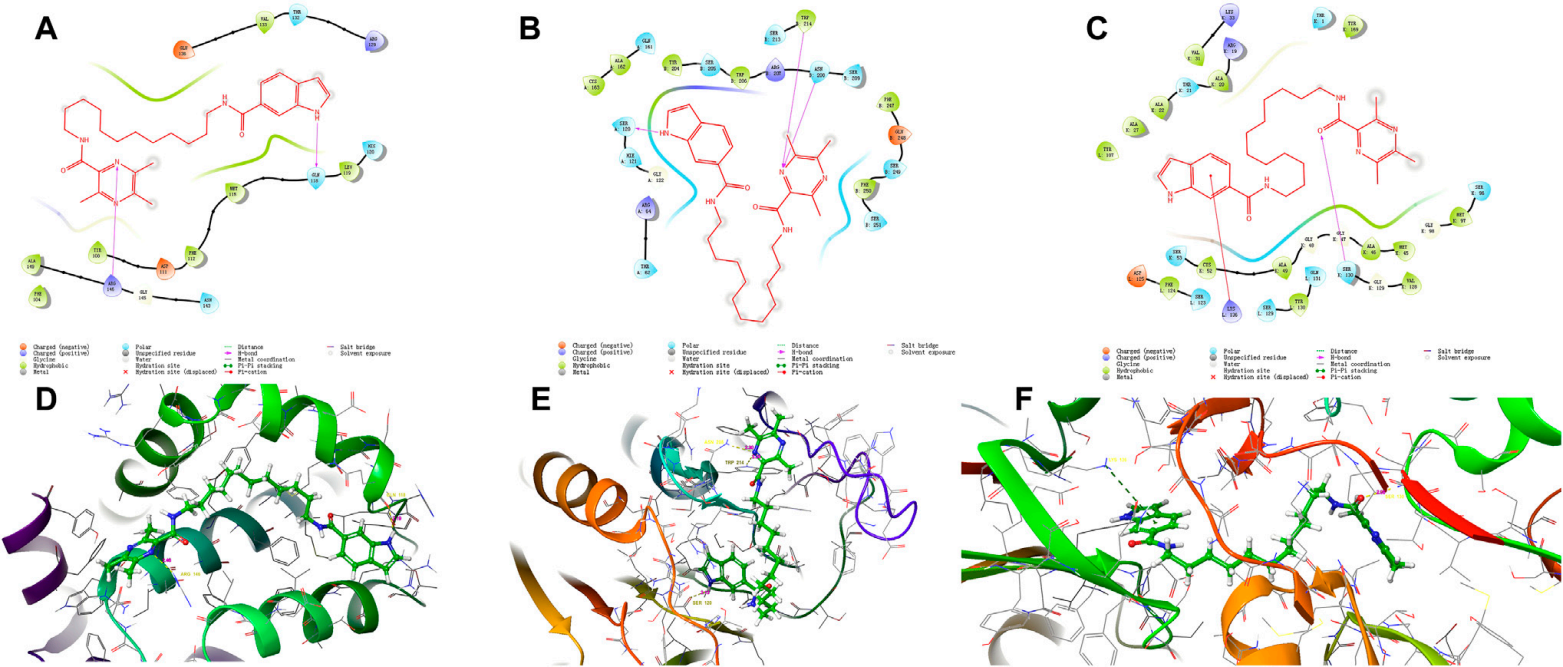
Visual presentation of the interaction of the crystal structure of compound **12–9** (shown as stick) with its docked pose of Bcl-2, CASP-3, and PSMB5 and (represented as a secondary structure in green) performed by the Schrodinger. [**(A–C)** represented as the ligand interaction diagram with Bcl-2, CASP-3, and PSMB5 respectively. **(D–F)** represented as binding interactions with Bcl-2, CASP-3, and PSMB5, respectively.].

Compound **12–9** and the reference control TMP were docked with the Bcl-2, CASP-3, and PSMB5 receptors using molecular docking protocols, and the binding modes between the ligand and protein were analyzed visually. The crystal structures of Bcl-2 (PDB ID: 6GL8), CASP-3 (PDB ID: 1NME), and PSMB5 (PDB ID: 5LEY) were downloaded into the Schrodinger Maestro platform and subjected to the protein preparation wizard using default parameters. The binding mode of compound **12–9** is shown in Figure 4. The docking scores of **12–9** and TMP with Bcl-2, CASP-3, and PSMB5 were −4.495 and −4.226, −5.410 and −4.331, and −7.925 and −5.509, respectively. The scoring functions indicated that **12–9** had a greater affinity with the targets than TMP, which was used as a reference. Visual inspection of the results in Figure 4 indicated that compound **12–9** was embedded into the binding pocket of each antitumor target protein. TMPH **12–9** was bound firmly at the active site of each target receptor by two conventional hydrogen bonds. In addition to these hydrogen bonds, non-bonding amino acid residues were surrounded by the ligand **12–9** and each target complex surface. These results indicated that the synthesized ligustrazine derivative **12–9** may elicit anticancer effects by activating the Bcl-2, CASP-3 apoptotic pathway, and PSMB5 proliferation pathway. Moreover, the cell-specific response of compound **12–9** can be partly explained by the human MDA-MB-231 breast carcinoma cells completely.

### Prediction of *In Silico* Drug-Like Properties

The prediction of drug-like properties is becoming highly desirable because most drugs in development fail in clinical trials. The drug-like properties of the 33 newly synthesized TMPH compounds were evaluated using the Schrodinger Canvas and ADMETlab 2.0 platforms. Also, 12 key molecular descriptors for the physicochemical, medicinal chemistry, and ADMET properties were calculated to evaluate the drug-like properties, as shown in Table 2. The MWs of most of the compounds were under 500 Da, except for six compounds (**8–12**, **12–3**, **12–5**, **12–6**, **12–10**, and **12–13**). The logP values of 10 compounds (**8–1**, **8–2**, **8–4**, **8–5**, **8–6**, **8–9**, **8–12**, **10–6**, **10–11**, and **10–12**) were <5. The 33 TMPH compounds had 2–9 hydrogen bond acceptors and 0–3 hydrogen bond donors. These results indicated that only 3 of the 33 TMPH compounds (**12–3**, **12–6**, and **12–13**) violated two rules out of Lipinski’s rule of five (nHA ≤ 10, nHD ≤ 5, MW ≤ 500, and logP ≤ 5). Except for the logP values, 78.79% (26/33) of the TMPH compounds met the other requirements in Lipinski’s rule of five. Therefore, the structural modification should be conducted to optimize the logP value. According to the Pfizer rule, compounds with a high logP value (>3) and low TPSA (<75) are likely to be toxic. None of the 33 TMPH compounds had logP values > 3 indicating acceptable toxicity. In addition, none of the 33 TMPH compounds conformed to the GSK rule (MW ≤ 400, logP ≤4). The T_1/2_ and CL values were in the range of 0.02–0.5 h and 3.51–4.92, respectively, suggesting that the compounds would be rapidly metabolized. The IGC_50_ values of the compounds were in the range of 4.39–5.4 and 4.22–5.91, implying low toxicity. These results suggested that the synthesized compounds had good drug-likeness, as shown in Table 2. This information might be useful for designing new drugs with favorable drug-like properties for the treatment of TNBC.

**TABLE 2:** Drug-like properties of 33 TMPH compounds.

Compound	nHA	nHD	TPSA	MW	LogP	ROF	PPB	VDss	CYP1A2I	CL	T1/2	IGC_50_
**8–1**	6	2	83.98	422.57	4.25	0	0.94	1.89	0.35	3.58	0.20	4.62
**8–2**	7	2	97.12	436.55	4.66	0	0.95	2.97	0.38	4.41	0.15	4.60
**8–3**	6	2	83.98	452.61	5.06	0	0.97	1.38	0.32	4.37	0.04	4.70
**8–4**	7	3	99.77	435.57	4.74	0	0.89	1.67	0.41	4.19	0.15	4.45
**8–5**	7	2	96.87	447.58	4.08	0	0.86	2.54	0.28	4.30	0.17	4.66
**8–6**	7	2	96.87	447.58	3.94	0	0.80	2.36	0.23	4.00	0.18	4.63
**8–9**	7	3	99.77	435.57	4.10	0	0.86	2.06	0.28	4.51	0.19	4.58
**8–10**	6	2	83.98	457.01	5.08	1	0.96	2.07	0.31	3.51	0.09	4.88
**8–11**	8	2	102.44	420.55	3.96	0	0.90	0.95	0.18	4.92	0.37	4.58
**8–12**	9	2	111.67	512.65	3.86	0	0.87	0.80	0.17	4.80	0.40	4.39
**8–13**	6	2	83.98	446.59	5.10	1	0.96	2.12	0.22	4.58	0.11	4.95
**10–1**	6	2	83.98	450.62	5.21	1	0.96	2.33	0.25	3.57	0.14	5.03
**10–2**	7	2	97.12	464.61	5.65	1	0.97	3.58	0.26	4.37	0.11	5.01
**10–3**	6	2	83.98	480.67	6.03	1	0.98	1.79	0.21	4.37	0.03	5.06
**10–4**	7	3	99.77	463.62	5.73	1	0.94	1.90	0.29	4.18	0.11	4.93
**10–5**	7	2	96.87	475.63	5.03	1	0.93	2.85	0.20	4.31	0.13	5.04
**10–6**	7	2	96.87	475.63	4.87	0	0.88	2.71	0.18	4.02	0.14	5.03
**10–9**	7	3	99.77	463.62	5.08	1	0.93	2.34	0.21	4.52	0.15	5.01
**10–10**	6	2	83.98	485.07	6.00	1	0.97	2.68	0.22	3.58	0.06	5.15
**10–11**	8	2	102.44	448.61	4.92	0	0.94	0.84	0.15	4.62	0.29	5.01
**10–12**	9	2	111.67	448.61	4.82	0	0.92	0.64	0.14	4.56	0.29	4.90
**10–13**	6	2	83.98	474.65	6.11	1	0.97	2.48	0.16	4.57	0.08	5.18
**12–1**	6	2	83.98	478.68	6.24	1	0.97	2.86	0.19	3.64	0.10	5.25
**12–2**	7	2	97.12	492.66	6.66	1	0.98	4.07	0.18	4.35	0.08	5.23
**12–3**	6	2	83.98	508.72	7.00	2	0.99	2.28	0.15	4.32	0.02	5.28
**12–4**	7	3	99.77	491.68	6.72	1	0.96	2.42	0.20	4.17	0.08	5.17
**12–5**	7	2	96.87	503.69	6.03	1	0.96	3.23	0.15	4.31	0.09	5.26
**12–6**	7	2	96.87	503.69	5.88	2	0.94	3.07	0.14	3.99	0.10	5.25
***12–9***	7	3	99.77	491.68	6.08	1	0.96	2.78	0.15	4.46	0.11	5.23
**12–10**	6	2	83.98	513.12	6.92	1	0.98	3.21	0.17	3.59	0.04	5.37
**12–11**	8	2	102.44	476.66	5.92	1	0.97	0.85	0.13	4.40	0.20	5.23
**12–12**	9	2	111.67	476.66	5.78	1	0.95	0.56	0.12	4.40	0.20	5.15
**12–13**	6	2	83.98	502.70	7.07	2	0.98	2.95	0.13	4.50	0.06	5.40
TMP	2	0	25.78	136.19	1.34	0	0.60	1.39	0.39	6.04	0.27	2.70

Note: nHA, number of hydrogen bond acceptors; nHD, number of hydrogen bond donors; TPSA, topological polar surface area; MW, molecular weight; logP, logarithm of the n-octanol/water distribution coefficient; PPB, plasma protein binding; VD, volume distribution; CL, clearance; T1/2, half-life; IGC_50_, 48 h *Tetrahymena pyriformis* IGC_50_.

## Conclusion

A series of 33 TMPH compounds consisting of a TMP derivative linked to a pharmaco-active heterocycle by a flexible alkyl chain were rationally designed and synthesized as anticancer agents that targeted PSMB5 with good IC_50_ values in the low micromolar range. Cell-based antiproliferative assays led to the identification of compound **12–9**, which had IC_50_ values ranging from 0.84 ± 0.02 to 12.37 ± 0.09 µM in the five tested cell lines. The antitumor effects of TMPH **12–9** were further evaluated by plate cloning, Hoechst 333 42 staining, and annexin V-FITC/PI staining. TMPH **12–9** inhibited the proliferation and apoptosis of breast cancer cells. Molecular docking of compound **12–9** into the active site of Bcl-2, CASP-3, and PSMB5 proteins was performed to explore the probable binding mode. Finally, the 33 newly synthesized compounds were predicted to have good drug-like properties in a theoretical study.

In conclusion, in the present study, TMPH derivatives were systematically designed and synthesized, and the antitumor proliferation and apoptotic activities, molecular docking, and prediction of drug-like properties were analyzed. The results showed that compound **12–9** had good antiproliferative and apoptosis-inducing effects in the human breast cancer MDA-MB-231 cell line and had good drug-like properties in a theoretical study. This study provides a foundation for the research and development of effective bioactive components from Chinese natural medicines as candidate antitumor drugs.

## Data Availability

The original contributions presented in the study are included in the article/Supplementary Material; further inquiries can be directed to the corresponding authors.
