# Supplementary material for: Synthesis and Discovery of Ligustrazine–Heterocycle Derivatives as Antitumor Agents
Source: Front Chem. 2022 Jul 25;10:941367. doi: 10.3389/fchem.2022.941367 (PMC9358002; doi:10.3389/fchem.2022.941367)
Supplement: Supplementary file 1 [file DataSheet1.docx]

Supplementary Material

### 1. Structure characterization results of 8-series TMPH compounds

***N*-(8-cinnamamidooctyl)-3,5,6-trimethylpyrazine-2-carboxamide (8-1)**：Pale white solid, yield：45%, m.p. 86.5–87.3℃; ^1^H NMR (400 MHz, CDCl_3_) δ 8.03 (s, 1H), 7.62 (d, *J* = 15.6 Hz, 1H), 7.47 (dd, *J* = 6.4, 2.8 Hz, 2H), 7.33 (dd, *J* = 4.9, 1.7 Hz, 3H), 6.46 (d, *J* = 15.6 Hz, 1H), 6.13 (s, 1H), 3.39 (td, *J* = 13.9, 6.8 Hz, 4H), 2.91 (s, 3H), 2.53 (d, *J* = 12.5 Hz, 6H), 1.66 – 1.51 (m, 4H), 1.36 (d, *J* = 19.8 Hz, 8H). ^13^C NMR (101 MHz, CDCl_3_) δ 166.10, 165.19, 154.07, 151.35, 147.65, 140.70, 139.19, 135.14, 129.61, 128.87, 127.83, 121.22, 39.88, 39.45, 31.54, 29.76, 29.25, 27.03, 26.99, 23.00, 22.79, 22.07, 21.49, 14.21. HRMS (ESI) m/z: calcd. for C_25_H_35_N_4_O_2_ [M + H]^+^ 423.2755, found 423.2742.

***N*-(8-(benzofuran-2-carboxamido)octyl)-3,5,6-trimethylpyrazine-2-carboxamide (8-2)** ：Pale white solid, yield：48%, m.p. 100.2–100.9℃; ^1^H NMR (400 MHz, CDCl_3_) δ 8.02 (s, 1H), 7.66 (d, *J* = 7.8 Hz, 1H), 7.44 (dt, *J* = 15.5, 8.4 Hz, 3H), 7.28 (t, *J* = 7.5 Hz, 1H), 6.72 (s, 1H), 3.45 (ddd, *J* = 23.2, 13.5, 6.8 Hz, 4H), 2.91 (s, 3H), 2.53 (d, *J* = 13.2 Hz, 6H), 1.64 (dd, *J* = 14.1, 6.9 Hz, 4H), 1.38 (s, 8H). ^13^C NMR (101 MHz, CDCl_3_) δ 165.03, 158.86, 154.72, 153.91, 151.27, 149.01, 147.48, 139.09, 127.70, 126.73, 123.65, 122.67, 111.69, 110.13, 110.03, 39.38, 39.33, 29.68, 29.64, 29.17, 26.97, 26.86, 22.89, 21.96, 21.37. HRMS (ESI) m/z: calcd. for C_25_H_33_N_4_O_3_ [M + H]^+^ 437.2547, found 437.2547.

***N*-(8-(benzo[b]thiophene-2-carboxamido)octyl)-3,5,6-trimethylpyrazine-2-carboxamide (8-3)** ：Pale white solid, yield：30%, m.p. 125.4–126.0℃; ^1^H NMR (400 MHz, CDCl_3_) δ 8.03 (s, 1H), 7.87 – 7.75 (m, 3H), 7.40 (pd, *J* = 7.1, 1.4 Hz, 2H), 6.45 (s, 1H), 3.44 (ddd, *J* = 18.9, 13.5, 6.8 Hz, 4H), 2.91 (s, 3H), 2.53 (d, *J* = 13.0 Hz, 6H), 1.69 – 1.55 (m, 4H), 1.36 (s, 8H). ^13^C NMR (101 MHz, CDCl_3_) δ 165.07, 162.32, 153.94, 151.25, 147.52, 140.78, 139.18, 139.08, 138.83, 126.19, 125.00, 124.96, 124.84, 122.67, 40.22, 39.32, 29.65, 29.59, 29.12, 26.92, 26.85, 22.89, 21.96, 21.38. HRMS (ESI) m/z: calcd. for C_25_H_33_N_4_O_2_S [M + H]^+^ 453.2319, found 453.2316.

***N*-(8-(3,5,6-trimethylpyrazine-2-carboxamido)octyl)-*1H*-indole-2-carboxamide (8-4)** ：Pale white solid, yield：50%, m.p. 126.4–127.2℃; ^1^H NMR (400 MHz, CDCl_3_) δ 9.87 (s, 1H), 8.03 (s, 1H), 7.62 (d, *J* = 8.0 Hz, 1H), 7.42 (t, *J* = 7.8 Hz, 1H), 7.26 (t, *J* = 7.6 Hz, 1H), 7.12 (t, *J* = 7.8 Hz, 1H), 6.86 (s, 1H), 6.45 (t, *J* = 5.5 Hz, 1H), 3.45 (ddd, *J* = 34.3, 13.6, 6.8 Hz, 4H), 2.91 (s, 3H), 2.52 (d, *J* = 15.2 Hz, 6H), 1.61 (dt, *J* = 15.5, 7.6 Hz, 4H), 1.34 (s, 8H). ^13^C NMR (101 MHz, CDCl_3_) δ 165.08, 161.79, 153.99, 151.25, 147.55, 139.04, 136.39, 130.95, 127.64, 124.28, 121.82, 120.50, 112.04, 101.88, 39.70, 39.31, 29.69, 29.62, 29.10, 29.08, 26.87, 26.82, 22.92, 21.98, 21.38. HRMS (ESI) m/z: calcd. for C_25_H_33_N_5_NaO_2_ [M + Na]^+^ 458.2526, found 458.2525.

***N*-(8-(3,5,6-trimethylpyrazine-2-carboxamido)octyl)quinoline-3-carboxamide** **(8-5)** ：Pale white solid, yield：42%, m.p.110.2–111.8℃; ^1^H NMR (400 MHz, CDCl_3_) δ 9.29 (d, *J* = 1.8 Hz, 1H), 8.61 (d, *J* = 1.3 Hz, 1H), 8.11 (t, *J* = 12.0 Hz, 1H), 8.02 (s, 1H), 7.92 – 7.71 (m, 2H), 7.59 (t, *J* = 7.4 Hz, 1H), 6.80 (s, 1H), 3.46 (ddd, *J* = 46.4, 13.5, 6.8 Hz, 4H), 2.89 (s, 3H), 2.52 (d, *J* = 13.5 Hz, 6H), 1.71 – 1.56 (m, 4H), 1.35 (s, 8H). ^13^C NMR (101 MHz, CDCl_3_) δ 165.69, 165.06, 153.97, 151.22, 149.11, 148.26, 147.53, 139.01, 135.53, 131.12, 129.30, 128.72, 127.44, 126.95, 40.29, 39.29, 29.64, 29.57, 29.10, 26.89, 22.89, 21.97, 21.39. HRMS (ESI) m/z: calcd. for C_26_H_34_N_5_O_2_ [M + H]^+^ 448.2707, found 448.2703.

***N*-(8-(3,5,6-trimethylpyrazine-2-carboxamido)octyl)quinoline-6-carboxamide** **(8-6)** ：Pale white solid, yield：36%, m.p. 109.6–110.2℃; ^1^H NMR (400 MHz, CDCl_3_) δ 8.95 (dd, *J* = 4.2, 1.7 Hz, 1H), 8.30 (d, *J* = 1.6 Hz, 1H), 8.18 (dd, *J* = 8.3, 1.2 Hz, 1H), 8.08 (dt, *J* = 8.8, 5.3 Hz, 2H), 8.02 (s, 1H), 7.43 (dd, *J* = 8.3, 4.2 Hz, 1H), 6.74 (s, 1H), 3.44 (ddd, *J* = 39.6, 13.5, 6.7 Hz, 4H), 2.89 (s, 3H), 2.51 (d, *J* = 13.6 Hz, 6H), 1.68 – 1.56 (m, 4H), 1.35 (s, 8H). ^13^C NMR (101 MHz, CDCl_3_) δ 166.94, 165.07, 153.97, 151.80, 151.15, 149.21, 147.56, 138.99, 136.97, 132.80, 129.77, 127.56, 127.28, 121.82, 40.31, 39.29, 29.63, 29.59, 29.14, 29.11, 26.92, 26.90, 22.88, 21.96, 21.38. HRMS (ESI) m/z: calcd. for C_26_H_34_N_5_O_2_ [M + H]^+^, 448.2707, found 448.2708.

***N*-(8-(3,5,6-trimethylpyrazine-2-carboxamido)octyl)-*1H*-indole-6-carboxamide** **(8-9)** ：Pale white solid, yield：52%, m.p. 119.3–120.5℃; ^1^H NMR (400 MHz, CDCl_3_ + CD_3_OD) δ 7.90 (s, 1H), 7.57 (d, *J* = 8.3 Hz, 1H), 7.37 (d, *J* = 8.2 Hz, 1H), 7.29 (d, *J* = 2.9 Hz, 1H), 6.49 (d, *J* = 2.8 Hz, 1H), 3.42 – 3.33 (m, 4H), 2.84 (s, 3H), 2.49 (d, *J* = 11.0 Hz, 6H), 1.61 – 1.53 (m, 4H), 1.32 (s, 8H). ^13^C NMR (101 MHz, CDCl_3_ + CD_3_OD) δ 169.46, 165.30, 154.12, 151.01, 148.06, 139.24, 135.69, 130.48, 127.39, 120.22, 117.44, 111.37, 101.96, 40.12, 39.35, 33.82, 29.57, 29.18, 26.93, 22.61, 21.73, 21.37. HRMS (ESI) m/z: calcd. for C_25_H_34_N_5_O_2_ [M + H]^+^, 436.2707, found 436.2703.

**(*E*)-*N*-(8-(3-(4-chlorophenyl)acrylamido)octyl)-3,5,6-trimethylpyrazine-2-carboxamide (8-10)** ：Pale white solid, yield：46%, m.p. 129.2–130.1℃; ^1^H NMR (400 MHz, CDCl_3_) δ 8.02 (s, 1H), 7.54 (d, *J* = 15.6 Hz, 1H), 7.36 (d, *J* = 8.4 Hz, 2H), 7.26 (d, *J* = 8.3 Hz, 2H), 6.52 – 6.35 (m, 2H), 3.37 (td, *J* = 14.0, 6.6 Hz, 4H), 2.89 (s, 3H), 2.51 (d, *J* = 13.0 Hz, 6H), 1.65 – 1.48 (m, 4H), 1.30 (s, 8H). ^13^C NMR (101 MHz, CDCl_3_) δ 165.77, 165.07, 154.00, 151.13, 147.63, 139.08, 139.00, 135.24, 133.51, 128.98, 128.86, 121.77, 39.78, 39.31, 29.63, 29.57, 29.12, 26.89, 22.88, 21.96, 21.40. HRMS (ESI) m/z: calcd. for C_25_H_34_ClN_4_O_2_ [M + H]^+^ 457.2365, found 457.2355.

**(*E*)-*N*-(8-(3-(3,4-dimethoxyphenyl)acrylamido)octyl)-3,5,6-trimethylpyrazine-2-carboxamide** **(8-11)** ：Pale white solid, yield：40%, m.p. 115.5–116.3℃; ^1^H NMR (400 MHz, CDCl_3_) δ 7.39 (d, *J* = 14.0 Hz, 1H), 7.06 – 6.93 (m, 2H), 6.79 (d, *J* = 8.2 Hz, 1H), 6.33 (d, *J* = 15.7 Hz, 1H), 4.35 (s, 2H), 3.80 (s, 6H), 3.33 – 3.18 (m, 4H), 2.76 (s, 3H), 2.46 (d, *J* = 4.0 Hz, 6H), 1.60 – 1.39 (m, 4H), 1.28 (s, 8H). ^13^C NMR (101 MHz, CDCl_3_) δ 166.63, 164.75, 153.23, 149.69, 149.67, 148.26, 147.65, 139.47, 138.78, 127.30, 121.10, 117.96, 110.42, 109.11, 54.91, 54.83, 38.75, 38.50, 28.77, 28.58, 28.47, 28.32, 26.05, 26.02, 21.20, 20.41, 20.19. HRMS (ESI) m/z: calcd. for C_27_H_39_N_4_O_4_ [M + H]^+^ 483.2966, found 483.2963.

**(*E*)-3,5,6-trimethyl-*N*-(8-(3-(3,4,5-trimethoxyphenyl)acrylamido)octyl)pyrazine-2-carboxamide** **(8-12)** ：Pale white solid, yield：55%, m.p. 122.8–123.2℃; ^1^H NMR (400 MHz, CDCl_3_ + CD_3_OD) δ 7.58 (d, *J* = 15.6 Hz, 1H), 6.83 (s, 2H), 6.65 (d, *J* = 15.7 Hz, 1H), 3.88 (d, *J* = 15.9 Hz, 9H), 3.40 (d, *J* = 25.9 Hz, 4H), 3.16 (s, 3H), 2.84 (d, *J* = 63.4 Hz, 6H), 1.63 (s, 4H), 1.37 (s, 8H). ^13^C NMR (101 MHz, CDCl_3_ + CD_3_OD) δ 167.98, 162.43, 155.09, 153.49, 153.45, 149.04, 145.35, 144.74, 142.59, 139.94, 130.41, 130.33, 118.37, 105.63, 61.04, 56.34, 40.67, 40.00, 39.88, 29.41, 29.22, 29.13, 27.00, 26.95, 21.33, 17.97, 17.65. HRMS (ESI) m/z: calcd. for C_28_H_41_N_4_O_5_ [M + H]^+^ 513.3071, found 513.3071.

***N*-(8-(2-naphthamido)octyl)-3,5,6-trimethylpyrazine-2-carboxamide (8-13)** ：Pale white solid, yield：42%, m.p. 130.4–131.7℃; ^1^H NMR (400 MHz, CDCl_3_ + CD_3_OD) δ 8.24 (d, *J* = 7.7 Hz, 1H), 7.89 (dd, *J* = 16.2, 7.3 Hz, 2H), 7.60 – 7.42 (m, 4H), 3.46 (m, 4H), 2.88 (d, *J* = 2.3 Hz, 3H), 2.62 – 2.46 (m, 6H), 1.67 (s, 4H), 1.40 (s, 8H). ^13^C NMR (101 MHz, CDCl_3_ + CD_3_OD) δ 169.40, 164.32, 153.17, 150.05, 147.17, 138.30, 133.72, 132.74, 129.53, 129.14, 127.40, 126.12, 125.49, 124.31, 123.95, 123.84, 39.09, 38.38, 28.78, 28.61, 28.27, 26.01, 21.57, 20.72, 20.41. HRMS (ESI) m/z: calcd. for C_27_H_35_N_4_O_2_ [M + H]^+^ 447.2755, found 447.2753.

### 2. Structure characterization results of 10-series TMPH compounds

***N*-(10-cinnamamidodecyl)-3,5,6-trimethylpyrazine-2-carboxamide** **(10-1)** ：Pale white solid, yield：42%, m.p. 92.4–93.2℃; ^1^H NMR (400 MHz, CDCl_3_) δ 8.02 (s, 1H), 7.62 (d, *J* = 15.6 Hz, 1H), 7.47 (dd, *J* = 6.4, 2.9 Hz, 2H), 7.37 – 7.26 (m, 3H), 6.45 (d, *J* = 15.6 Hz, 1H), 6.12 (s, 1H), 3.39 (dt, *J* = 13.4, 6.9 Hz, 4H), 2.91 (s, 3H), 2.53 (d, *J* = 12.8 Hz, 6H), 1.66 – 1.51 (m, 4H), 1.31–1.21 (m, 12H). ^13^C NMR (101 MHz, CDCl_3_) δ 166.01, 165.08, 153.97, 151.25, 147.56, 140.60, 139.12, 135.05, 129.52, 128.79, 127.75, 121.13, 39.84, 39.41, 30.36, 29.71, 29.42, 29.27, 27.03, 26.98, 22.92, 21.99, 21.42. HRMS (ESI) m/z: calcd. for C_27_H_39_N_4_O_2_ [M + H]^+^ 451.3068, found 451.3066.

***N*-(10-(benzofuran-2-carboxamido)decyl)-3,5,6-trimethylpyrazine-2-carboxamide (10-2)** ：Pale white solid, yield：44%, m.p. 103.2–104.1℃; ^1^H NMR (400 MHz, CDCl_3_) δ 8.02 (s, 1H), 7.66 (d, *J* = 7.8 Hz, 1H), 7.56 – 7.34 (m, 3H), 7.32 – 7.25 (m, 1H), 6.70 (s, 1H), 3.45 (ddd, *J* = 25.0, 13.5, 6.9 Hz, 4H), 2.92 (s, 3H), 2.53 (d, *J* = 13.6 Hz, 6H), 1.71 – 1.54 (m, 4H), 1.44 – 1.27 (m, 12H). ^13^C NMR (101 MHz, CDCl_3_) δ 165.01, 158.85, 154.72, 153.89, 151.29, 149.03, 147.46, 139.12, 127.72, 126.73, 123.65, 122.68, 111.68, 110.13, 110.03, 39.42, 39.38, 29.71, 29.67, 29.43, 29.42, 29.28, 29.25, 27.04, 26.93, 22.89, 21.96, 21.38. HRMS (ESI) m/z: calcd. for C_27_H_37_N_4_O_3_ [M + H]^+^ 465.2860, found 465.2861.

***N*-(10-(benzo[b]thiophene-2-carboxamido)decyl)-3,5,6-trimethylpyrazine-2-carboxamide (10-3)** ：Pale white solid, yield：32%, m.p. 128.7–129.2.2℃; ^1^H NMR (400 MHz, CDCl_3_) δ 8.02 (s, 1H), 7.88 – 7.74 (m, 3H), 7.44 – 7.34 (m, 2H), 6.48 (s, 1H), 3.50 – 3.36 (m, 4H), 2.91 (s, 3H), 2.53 (d, *J* = 13.3 Hz, 6H), 1.68 – 1.56 (m, 4H), 1.36–1.26 (m, 12H). ^13^C NMR (101 MHz, CDCl_3_) δ 165.05, 162.31, 153.92, 151.25, 147.50, 140.78, 139.18, 139.11, 138.86, 126.18, 124.99, 124.95, 124.83, 122.67, 40.27, 39.38, 29.69, 29.64, 29.38, 29.23, 27.00, 26.93, 22.88, 21.95, 21.37. HRMS (ESI) m/z: calcd. for C_27_H_37_N_4_O_2_S [M + H]^+^ 481.2632, found 481.2634.

***N*-(10-(3,5,6-trimethylpyrazine-2-carboxamido)decyl)-*1H*-indole-2-carboxamide (10-4)** ：Pale white solid, yield：45%, m.p. 129.1–130.8℃; ^1^H NMR (400 MHz, CDCl_3_) δ 10.13 (s, 1H), 8.04 (s, 1H), 7.63 (d, *J* = 8.0 Hz, 1H), 7.46 (d, *J* = 8.3 Hz, 1H), 7.27 (t, *J* = 7.4 Hz, 1H), 7.12 (t, *J* = 7.3 Hz, 1H), 6.89 (s, 1H), 6.56 (s, 1H), 3.47 (ddd, *J* = 37.1, 13.5, 6.8 Hz, 4H), 2.93 (s, 3H), 2.53 (d, *J* = 14.9 Hz, 6H), 1.71 – 1.55 (m, 4H), 1.34 (dd, *J* = 20.7, 10.9 Hz, 12H). ^13^C NMR (101 MHz, CDCl_3_) δ 165.09, 161.87, 153.94, 151.26, 147.51, 139.14, 136.51, 131.05, 127.68, 124.23, 121.80, 120.47, 112.09, 101.90, 39.81, 39.40, 29.76, 29.67, 29.36, 29.21, 26.98, 26.93, 22.88, 21.94, 21.35. HRMS (ESI) m/z: calcd. for C_27_H_38_N_5_O_2_ [M + H]^+^ 464.3020, found 464.3017.

***N*-(10-(3,5,6-trimethylpyrazine-2-carboxamido)decyl)quinoline-3-carboxamide** **(10-5)** ：Pale white solid, yield：40%, m.p. 113.7–114.5℃; ^1^H NMR (400 MHz, CDCl_3_) δ 9.28 (d, *J* = 2.2 Hz, 1H), 8.60 (d, *J* = 1.9 Hz, 1H), 8.11 (t, *J* = 8.9 Hz, 1H), 8.02 (s, 1H), 7.91 – 7.72 (m, 2H), 7.63 – 7.54 (m, 1H), 6.76 (s, 1H), 3.46 (ddd, *J* = 46.8, 13.4, 6.9 Hz, 4H), 2.89 (s, 3H), 2.52 (d, *J* = 14.0 Hz, 6H), 1.71 – 1.55 (m, 4H), 1.35 – 1.21 (m, 12H). ^13^C NMR (101 MHz, CDCl_3_) δ 165.72, 165.06, 153.92, 151.23, 149.18, 148.28, 147.50, 139.09, 135.46, 131.06, 129.35, 128.70, 127.45, 127.41, 126.97, 40.35, 39.37, 30.35, 29.68, 29.64, 29.36, 29.22, 29.21, 26.97, 22.87, 21.95, 21.37. HRMS (ESI) m/z: calcd. for C_28_H_38_N_5_O_2_ [M + H]^+^ 476.3020, found 476.3022.

***N*-(10-(3,5,6-trimethylpyrazine-2-carboxamido)decyl)quinoline-6-carboxamide** **(10-6)** ：Pale white solid, yield：42%, m.p. 113.1–114.6℃; ^1^H NMR (400 MHz, CDCl_3_) δ 8.95 (d, *J* = 2.6 Hz, 1H), 8.30 (d, *J* = 1.4 Hz, 1H), 8.18 (d, *J* = 7.6 Hz, 1H), 8.07 (dt, *J* = 8.8, 5.3 Hz, 2H), 8.01 (s, 1H), 7.43 (dd, *J* = 8.3, 4.2 Hz, 1H), 6.69 (s, 1H), 3.45 (ddd, *J* = 40.6, 13.5, 6.9 Hz, 4H), 2.89 (s, 3H), 2.51 (d, *J* = 13.7 Hz, 6H), 1.70 – 1.55 (m, 4H), 1.37–1.24 (m, 12H). ^13^C NMR (101 MHz, CDCl_3_) δ 166.90, 165.05, 153.92, 151.82, 151.21, 149.29, 147.50, 139.09, 136.92, 132.87, 129.86, 127.59, 127.52, 127.21, 121.83, 40.37, 39.36, 30.34, 29.68, 29.39, 29.25, 29.22, 26.99, 22.87, 21.95, 21.37. HRMS (ESI) m/z: calcd. for C_28_H_38_N_5_O_2_ [M + H]^+^ 476.3020, found 476.3017.

***N*-(10-(3,5,6-trimethylpyrazine-2-carboxamido)decyl)-*1H*-indole-6-carboxamide** **(10-9)** ：Pale white solid, yield：50%, m.p. 122.3–123.6℃; ^1^H NMR (400 MHz, CDCl_3_) δ 9.47 (s, 1H), 8.04 (d, *J* = 5.7 Hz, 2H), 7.63 (d, *J* = 8.3 Hz, 1H), 7.43 (dd, *J* = 8.3, 1.4 Hz, 1H), 7.37 – 7.30 (m, 1H), 6.56 (s, 1H), 6.35 (s, 1H), 3.45 (ddd, *J* = 23.1, 13.4, 6.9 Hz, 4H), 2.92 (s, 3H), 2.53 (d, *J* = 13.7 Hz, 6H), 1.70 – 1.55 (m, 4H), 1.40 – 1.25 (m, 12H). ^13^C NMR (101 MHz, CDCl_3_) δ 168.66, 165.09, 153.94, 151.23, 147.54, 139.12, 135.67, 130.38, 128.15, 127.17, 120.30, 117.46, 111.42, 102.36, 40.16, 39.42, 29.75, 29.68, 29.37, 29.35, 29.22, 27.01, 26.94, 22.88, 21.94, 21.37. HRMS (ESI) m/z: calcd. for C_27_H_38_N_5_O_2_ [M + H]^+^464.3020, found 464.3018.

**(*E*)-*N*-(10-(3-(4-chlorophenyl)acrylamido)decyl)-3,5,6-trimethylpyrazine-2-carboxamide** **(10-10)** ：Pale white solid, yield：43%, m.p. 133.3–134.7℃; ^1^H NMR (400 MHz, CDCl_3_) δ 8.02 (s, 1H), 7.55 (d, *J* = 15.6 Hz, 1H), 7.34 (dd, *J* = 37.4, 8.6 Hz, 4H), 6.42 (d, *J* = 15.6 Hz, 1H), 6.15 (s, 1H), 3.39 (ddd, *J* = 17.2, 13.6, 6.9 Hz, 4H), 2.90 (s, 3H), 2.53 (d, *J* = 13.1 Hz, 6H), 1.66 – 1.50 (m, 4H), 1.37 – 1.23 (m, 12H). ^13^C NMR (101 MHz, CDCl_3_) δ 165.66, 165.07, 153.96, 151.23, 147.53, 139.19, 139.09, 135.31, 133.55, 129.01, 128.88, 121.73, 39.84, 39.38, 29.69, 29.65, 29.38, 29.37, 29.22, 29.21, 26.99, 26.94, 22.89, 21.96, 21.38. HRMS (ESI) m/z: calcd. for C_27_H_38_ClN_4_O_2_ [M + H]^+^ 485.2678, found 485.2675.

**(*E*)-*N*-(10-(3-(3,4-dimethoxyphenyl)acrylamido)decyl)-3,5,6-trimethylpyrazine-2-carboxamide (10-11)** ：Pale white solid, yield：45%, m.p. 119.0–120.6℃; ^1^H NMR (400 MHz, CDCl_3_) δ 8.01 (s, 1H), 7.54 (d, *J* = 15.5 Hz, 1H), 7.09 – 6.96 (m, 2H), 6.82 (d, *J* = 8.3 Hz, 1H), 6.31 (d, *J* = 15.5 Hz, 1H), 5.97 (s, 1H), 3.87 (d, *J* = 7.3 Hz, 6H), 3.39 (ddd, *J* = 17.7, 13.5, 6.9 Hz, 4H), 2.90 (s, 3H), 2.52 (d, *J* = 13.1 Hz, 6H), 1.66 – 1.50 (m, 4H), 1.26 (dd, *J* = 31.1, 14.2 Hz, 12H). ^13^C NMR (101 MHz, CDCl_3_) δ 166.18, 165.05, 153.94, 151.24, 150.53, 149.18, 147.52, 140.43, 139.10, 128.04, 121.79, 119.01, 111.21, 109.88, 55.95, 55.87, 39.77, 39.38, 29.73, 29.70, 29.40, 29.25, 29.23, 27.00, 26.95, 22.89, 21.96, 21.38. HRMS (ESI) m/z: calcd. for C_29_H_43_N_4_O_4_ [M + H]^+^ 511.3279, found 511.3279.

**(*E*)-3,5,6-trimethyl-*N*-(10-(3-(3,4,5-trimethoxyphenyl)acrylamido)decyl)pyrazine-2-carboxamide** **(10-12)** ：Pale white solid, yield：48%, m.p. 125.3–126.7℃; ^1^H NMR (400 MHz, CDCl_3_) δ 8.02 (s, 1H), 7.52 (d, *J* = 15.5 Hz, 1H), 6.70 (s, 2H), 6.35 (d, *J* = 15.5 Hz, 1H), 6.00 (s, 1H), 3.85 (d, *J* = 4.0 Hz, 9H), 3.39 (dt, *J* = 13.2, 6.9 Hz, 4H), 2.90 (s, 3H), 2.53 (d, *J* = 13.3 Hz, 6H), 1.67 – 1.50 (m, 4H), 1.30 (d, *J* = 21.2 Hz, 12H). ^13^C NMR (101 MHz, CDCl_3_) δ 165.87, 165.05, 153.96, 153.41, 151.24, 147.52, 140.53, 139.61, 139.09, 130.59, 120.46, 105.04, 60.92, 56.14, 39.80, 39.37, 29.70, 29.39, 29.24, 29.22, 26.99, 26.95, 22.89, 21.96, 21.37. HRMS (ESI) m/z: calcd. for C_30_H_45_N_4_O_5_ [M + H]^+^ 541.3384, found 541.3385.

**(*E*)-3,5,6-trimethyl-*N*-(10-(3-(3,4,5-trimethoxyphenyl)acrylamido)decyl)pyrazine-2-carboxamide** **(10-13)** ：Pale white solid, yield：40%, m.p. 137.6–138.2℃; ^1^H NMR (400 MHz, CDCl_3_ + CD_3_OD) δ 8.23 (d, *J* = 8.0 Hz, 1H), 7.87 (dd, *J* = 14.7, 8.0 Hz, 2H), 7.60 – 7.37 (m, 4H), 3.39 (s, 4H), 2.87 (s, 3H), 2.53 (d, *J* = 9.7 Hz, 6H), 1.64 (s, 4H), 1.34 (dd, *J* = 32.1, 21.0 Hz, 12H). ^13^C NMR (101 MHz, CDCl_3_ + CD_3_OD) δ 169.30, 164.26, 153.11, 150.04, 147.09, 138.28, 133.75, 132.71, 129.47, 127.37, 126.07, 125.45, 124.34, 123.93, 123.81, 39.11, 38.40, 28.63, 28.54, 28.35, 26.09, 21.61, 20.74, 20.41. HRMS (ESI) m/z: calcd. for C_29_H_39_N_4_O_2_ [M + H]^+^ 475.3068, found 475.3066.

### 3. Structure characterization results of 12-series TMPH compounds

***N*-(12-cinnamamidododecyl)-3,5,6-trimethylpyrazine-2-carboxamide** **(12-1)** ：Pale white solid, yield：40%, m.p. 95.5–96.9℃; ^1^H NMR (400 MHz, CDCl_3_) δ 8.02 (s, 1H), 7.62 (d, *J* = 15.6 Hz, 1H), 7.47 (dd, *J* = 6.5, 2.9 Hz, 1H), 7.38 – 7.26 (m, 3H), 6.44 (dd, *J* = 15.6, 5.2 Hz, 1H), 6.06 (s, 1H), 3.40 (dt, *J* = 15.1, 6.9 Hz, 4H), 2.91 (s, 3H), 2.53 (d, *J* = 13.4 Hz, 6H), 1.66 – 1.53 (m, 4H), 1.37 – 1.23 (m, 16H). ^13^C NMR (101 MHz, CDCl_3_) δ 165.93, 165.00, 153.93, 151.21, 147.53, 140.56, 139.05, 134.98, 129.48, 128.74, 127.70, 121.04, 39.81, 39.38, 29.68, 29.53, 29.49, 29.32, 29.30, 29.28, 27.06, 27.04, 26.97, 22.91, 21.97, 21.40. HRMS (ESI) m/z: calcd. for C_29_H_42_N_4_O_2_ [M + Na]^+^ 501.3200, found 501.3190.

***N*-(12-(benzofuran-2-carboxamido)dodecyl)-3,5,6-trimethylpyrazine-2-carboxamide (12-2)** ：Pale white solid, yield：42%, m.p. 107.3–108.0℃; ^1^H NMR (400 MHz, CDCl_3_ + CD_3_OD) δ 7.67 (d, *J* = 7.4 Hz, 1H), 7.53 – 7.35 (m, 3H), 7.29 (t, *J* = 7.5 Hz, 1H), 3.43 (dt, *J* = 21.2, 7.2 Hz, 4H), 2.88 (s, 3H), 2.54 (d, *J* = 9.1 Hz, 6H), 1.64 (dd, *J* = 15.9, 7.8 Hz, 4H), 1.30 (dd, *J* = 22.3, 13.4 Hz, 16H). ^13^C NMR (101 MHz, CDCl_3_ + CD_3_OD) δ 164.26, 158.44, 153.84, 153.09, 149.94, 147.61, 147.16, 138.30, 126.62, 125.95, 122.75, 121.75, 110.72, 109.40, 38.47, 38.39, 28.55, 28.53, 28.32, 26.05, 25.97, 21.45, 20.60, 20.31. HRMS (ESI) m/z: calcd. for C_29_H_41_N_4_O_3_ [M + H]^+^, 493.3173, found 493.3172.

***N*-(12-(benzofuran-2-carboxamido)dodecyl)-3,5,6-trimethylpyrazine-2-carboxamide (12-3)** ：Pale white solid, yield：28%, m.p. 132.5–133.1℃; ^1^H NMR (400 MHz, CDCl_3_ + CD_3_OD) δ 7.84 (d, *J* = 5.3 Hz, 3H), 7.46 – 7.36 (m, 2H), 3.48 – 3.38 (m, 3H), 2.88 (d, *J* = 4.9 Hz, 3H), 2.54 (d, *J* = 7.2 Hz, 6H), 1.63 (s, 4H), 1.31 (d, *J* = 23.8 Hz, 16H). ^13^C NMR (101 MHz, CDCl_3_ + CD_3_OD) δ 164.25, 162.07, 153.10, 149.91, 147.22, 139.90, 138.29, 125.23, 124.13, 123.84, 121.64, 39.24, 38.40, 28.54, 28.34, 26.03, 21.43, 20.58, 20.31. HRMS (ESI) m/z: calcd. for C_29_H_41_N_4_O_2_S [M + H]^+^ 509.2945, found 509.2947.

***N*-(12-(3,5,6-trimethylpyrazine-2-carboxamido)dodecyl)-*1H*-indole-2-carboxamide (12-4)** ：Pale white solid, yield：42%, m.p. 133.8–134.7℃; ^1^H NMR (400 MHz, CDCl_3_ + CD_3_OD) δ 7.56 (d, *J* = 8.0 Hz, 1H), 7.37 (d, *J* = 8.3 Hz, 1H), 7.20 (t, *J* = 7.2 Hz, 1H), 7.05 (t, *J* = 7.5 Hz, 1H), 6.90 (s, 1H), 3.36 (dt, *J* = 14.8, 7.4 Hz, 4H), 2.83 (s, 3H), 2.47 (d, *J* = 10.0 Hz, 6H), 1.55 (dd, *J* = 13.9, 6.9 Hz, 4H), 1.29 – 1.12 (m, 16H). ^13^C NMR (101 MHz, CDCl_3_ + CD_3_OD) δ 165.33, 162.39, 154.15, 151.00, 148.19, 139.35, 136.61, 130.99, 127.62, 124.28, 121.86, 120.42, 112.10, 102.95, 39.81, 39.45, 29.66, 29.60, 29.41, 29.36, 27.09, 22.55, 21.68, 21.38. HRMS (ESI) m/z: calcd. for C_29_H_42_N_5_O_2_ [M + H]^+^, 492.3333, found 492.3333.

***N*-(12-(3,5,6-trimethylpyrazine-2-carboxamido)dodecyl)quinoline-3-carboxamide** **(12-5)** ：Pale white solid, yield：35%, m.p. 115.8–116.9℃; ^1^H NMR (400 MHz, CDCl_3_ + CD_3_OD) δ 9.26 (s, 1H), 8.71 (s, 1H), 8.11 (d, *J* = 8.3 Hz, 1H), 7.93 (d, *J* = 8.1 Hz, 1H), 7.83 (d, *J* = 7.0 Hz, 1H), 7.73 – 7.58 (m, 1H), 3.54 – 3.39 (m, 4H), 2.90 (s, 3H), 2.55 (d, *J* = 9.8 Hz, 6H), 1.73 – 1.63 (m, 4H), 1.39 – 1.26 (m, 16H). ^13^C NMR (101 MHz, CDCl_3_ + CD_3_OD) δ 165.84, 165.16, 154.05, 151.06, 148.63, 148.22, 147.93, 139.18, 136.31, 131.36, 128.90, 128.72, 127.58, 127.53, 127.14, 40.35, 39.36, 29.73, 29.61, 29.55, 29.37, 29.32, 27.09, 27.06, 22.67, 21.78, 21.39. HRMS (ESI) m/z: calcd. for C_30_H_42_N_5_O_2_ [M + H]^+^ 504.3333, found 504.3336.

***N*-(12-(3,5,6-trimethylpyrazine-2-carboxamido)dodecyl)quinoline-6-carboxamide (12-6)** ：Pale white solid, yield：40%, m.p. 116.8–117.4℃; ^1^H NMR (400 MHz, CDCl_3_ + CD_3_OD) δ 8.91 (d, *J* = 2.2 Hz, 1H), 8.45 – 8.23 (m, 2H), 8.11 (q, *J* = 8.7 Hz, 2H), 7.52 (d, *J* = 6.0 Hz, 1H), 3.44 (dt, *J* = 26.0, 6.4 Hz, 4H), 2.87 (s, 3H), 2.55 (d, *J* = 5.6 Hz, 6H), 1.64 (dt, *J* = 21.4, 6.9 Hz, 4H), 1.32 (d, *J* = 26.3 Hz, 16H). ^13^C NMR (101 MHz, CDCl_3_ + CD_3_OD) δ 166.66, 164.47, 153.16, 150.54, 149.78, 147.70, 147.39, 138.50, 136.98, 132.02, 127.82, 126.95, 126.85, 121.04, 39.46, 38.48, 28.74, 28.60, 28.56, 28.42, 28.38, 26.16, 26.11, 21.37, 21.33, 20.55, 20.51, 20.30, 20.26. HRMS (ESI) m/z: calcd. for C_30_H_42_N_5_O_2_ [M + H]^+^ 504.3333, found 504.3331.

***N*-(12-(3,5,6-trimethylpyrazine-2-carboxamido)dodecyl)-*1H*-indole-6-carboxamide (12-9)** ：Pale white solid, yield：45%, m.p. 125.8–127.1℃; ^1^H NMR (400 MHz, CDCl_3_) δ 9.59 (s, 1H), 8.09 – 7.96 (m, 2H), 7.61 (d, *J* = 8.3 Hz, 1H), 7.42 (dd, *J* = 8.3, 1.3 Hz, 1H), 7.34 – 7.28 (m, 1H), 6.53 (s, 1H), 6.37 (t, *J* = 5.4 Hz, 1H), 3.43 (ddd, *J* = 20.5, 13.4, 6.9 Hz, 4H), 2.91 (s, 3H), 2.52 (d, *J* = 13.6 Hz, 6H), 1.67 – 1.53 (m, 4H), 1.38 – 1.22 (m, 16H). ^13^C NMR (101 MHz, CDCl_3_) δ 168.69, 165.06, 153.93, 151.19, 147.57, 139.09, 135.65, 130.36, 128.02, 127.24, 120.26, 117.39, 111.47, 102.28, 40.21, 39.41, 29.76, 29.68, 29.48, 29.28, 27.04, 27.01, 22.88, 21.94, 21.38. HRMS (ESI) m/z: calcd. for C_29_H_42_N_5_O_2_ [M + H]^+^ 492.3333, found 492.3331.

**(*E*)-*N*-(12-(3-(4-chlorophenyl)acrylamido)dodecyl)-3,5,6-trimethylpyrazine-2-carboxamide** **(12-10)** ：Pale white solid, yield：46%, m.p. 137.7–138.3℃; ^1^H NMR (400 MHz, CDCl_3_ + CD_3_OD) δ 7.52 (d, *J* = 15.7 Hz, 1H), 7.47 – 7.38 (m, 2H), 7.31 (dd, *J* = 8.4, 1.4 Hz, 2H), 6.46 (dd, *J* = 15.7, 1.4 Hz, 1H), 3.37 (dt, *J* = 32.6, 7.0 Hz, 4H), 2.88 (s, 3H), 2.54 (d, *J* = 9.1 Hz, 6H), 1.67 – 1.52 (m, 4H), 1.37 – 1.23 (m, 16H). ^13^C NMR (101 MHz, CDCl_3_ + CD_3_OD) δ 166.26, 165.22, 154.11, 151.07, 148.03, 139.30, 139.24, 135.38, 133.56, 129.06, 128.98, 121.52, 39.81, 39.39, 29.62, 29.55, 29.36, 29.32, 27.07, 27.03, 22.62, 21.75, 21.40. HRMS (ESI) m/z: calcd. for C_29_H_42_ClN_4_O_2_ [M + H]^+^ 513.2991, found 513.2993.

**(*E*)-*N*-(12-(3-(3,4-dimethoxyphenyl)acrylamido)dodecyl)-3,5,6-trimethylpyrazine-2-carboxamide** **(12-11)** ：Pale white solid, yield：42%, m.p. 122.1–122.7℃; ^1^H NMR (400 MHz, CDCl_3_ + CD_3_OD) δ 7.48 (d, *J* = 15.7 Hz, 1H), 7.12 (d, *J* = 9.1 Hz, 2H), 6.91 (d, *J* = 8.1 Hz, 1H), 6.44 (d, *J* = 15.7 Hz, 1H), 3.91 (d, J = 1.7 Hz, 6H), 3.42 – 3.30 (m, 4H), 2.84 (s, 3H), 2.56 (d, *J* = 1.9 Hz, 6H), 1.60 (ddd, *J* = 26.9, 13.5, 6.7 Hz, 4H), 1.32 (dd, *J* = 27.9, 8.8 Hz, 16H). ^13^C NMR (101 MHz, CDCl_3_ + CD_3_OD) δ 166.85, 164.98, 153.29, 149.82, 149.64, 148.41, 147.85, 139.56, 139.11, 127.44, 121.23, 118.01, 110.56, 109.24, 54.91, 54.86, 38.87, 38.64, 28.74, 28.67, 28.59, 28.54, 28.50, 26.20, 21.10, 20.34, 20.12. HRMS (ESI) m/z: calcd. for C_31_H_47_N_4_O_4_ [M + H]^+^ 539.3592, found 539.3589.

**(*E*)-3,5,6-trimethyl-*N*-(12-(3-(3,4,5-trimethoxyphenyl)acrylamido)dodecyl)pyrazine-2-carboxamide** **(12-12)** ：Pale white solid, yield：40%, m.p. 129.3–130.2℃; ^1^H NMR (400 MHz, CDCl_3_ + CD_3_OD) δ 7.49 (d, *J* = 15.6 Hz, 1H), 6.76 (d, *J* = 2.8 Hz, 2H), 6.39 (d, *J* = 15.6 Hz, 1H), 3.87 (dd, *J* = 6.4, 3.1 Hz, 9H), 3.37 (dt, *J* = 28.9, 7.1 Hz, 4H), 2.89 (d, *J* = 2.4 Hz, 3H), 2.55 (dd, *J* = 9.4, 2.5 Hz, 6H), 1.67 – 1.52 (m, 4H), 1.28 (s, 16H). ^13^C NMR (101 MHz, CDCl_3_ + CD_3_OD) δ 166.59, 165.27, 154.15, 153.41, 151.07, 148.11, 140.69, 139.47, 139.29, 130.76, 120.33, 105.08, 61.00, 56.17, 39.81, 39.42, 29.59, 29.40, 29.35, 27.09, 27.06, 22.59, 21.73, 21.40. HRMS (ESI) m/z: calcd. for C_32_H_49_N_4_O_5_ [M + H]^+^ 569.3697, found 569.3697.

***N*-(12-(2-naphthamido)dodecyl)-3,5,6-trimethylpyrazine-2-carboxamide** **(12-13)** ：Pale white solid, yield：36%, m.p. 141.7–142.5℃; ^1^H NMR (400 MHz, CDCl_3_ + CD_3_OD) δ 8.21 (d, *J* = 8.1 Hz, 1H), 7.84 (dd, *J* = 15.7, 8.0 Hz, 2H), 7.50 (dt, *J* = 21.5, 6.9 Hz, 3H), 7.44 – 7.37 (m, 1H), 3.42 (dt, *J* = 44.2, 7.1 Hz, 4H), 2.84 (s, 3H), 2.50 (d, *J* = 10.2 Hz, 6H), 1.65 – 1.55 (m, 4H), 1.27 (t, *J* = 18.1 Hz, 16H). ^13^C NMR (101 MHz, CDCl_3_ + CD_3_OD) δ 169.78, 165.03, 153.98, 151.22, 147.71, 139.12, 134.83, 133.69, 130.46, 130.11, 128.32, 127.08, 126.43, 125.36, 124.79, 124.76, 40.03, 39.30, 29.67, 29.65, 29.54, 29.32, 27.06, 27.02, 22.78, 21.87, 21.40. HRMS (ESI) m/z: calcd. for C_31_H_43_N_4_O_2_ [M + H]^+^ 503.3381, found 503.3381
